# Modality-general and modality-specific processes in hallucinations

**DOI:** 10.1017/S0033291719002496

**Published:** 2019-09-18

**Authors:** Charles Fernyhough

**Affiliations:** Durham University, Durham, UK

**Keywords:** Auditory hallucination, functional systems, Luria, multimodal hallucination, visual hallucination

## Abstract

There is a growing recognition in psychosis research of the importance of hallucinations in modalities other than the auditory. This has focused attention on cognitive and neural processes that might be shared by, and which might contribute distinctly to, hallucinations in different modalities. In this article, I address some issues around the modality-generality of cognitive and neural processes in hallucinations, including the role of perceptual and reality-monitoring systems, top-down and bottom-up processes in relation to the psychological and neural substrates of hallucinations, and the phenomenon of simultaneous multimodal hallucinations of the same entity. I suggest that a functional systems approach, inspired by some neglected aspects of the writings of A. R. Luria, can help us to understand patterns of hallucinatory experience across modalities and across clinical and non-clinical groups. Understanding the interplay between modality-general and modality-specific processes may bear fruit for improved diagnosis and therapeutic approaches to dealing with distressing hallucinations.

## Introduction

Contributing significantly to the distress of psychotic illness and increasingly recognized as part of the healthcare burden in other disorders, hallucinations cut across nosological boundaries with limited diagnostic specificity (Waters and Fernyhough, 2017). While the understanding of auditory hallucinations (AHs; ‘hearing voices’) has progressed in recent decades, non-auditory modalities, such as visual, tactile, and olfactory, have tended to be neglected in empirical psychosis research (Langdon *et al*., 2011; Waters *et al*., 2014; McCarthy-Jones *et al*., 2017). While this is likely due to their lower frequency, it is known that visual hallucinations (VHs) are reasonably common in psychotic disorders, with one recent review putting their incidence at 27% (weighted mean prevalence; Waters *et al*., 2014). Although individuals often experience hallucinations in more than one modality, there is only limited information on hallucinations in which the same entity is perceived at the same time in more than one modality: so-called ‘fused’ or simultaneous multimodal hallucinations (Goodwin *et al*., 1971; Hoffman and Varanko, 2006).

This raises questions about the extent to which the processes underlying hallucination are modality-general – liable to affect processing in different sensory modalities simultaneously – or specific to modalities of perception. Theoretical models of hallucinations in a particular modality (such as the inner speech misattribution model of AH; see below) may prove relevant to understanding hallucinations in other modalities, or they may turn out to be modality-specific. Although research into other modalities of hallucination lags behind research into AH, translating concepts from one modality to another (such as the role of deficits in peripheral sensory systems, as in eye disease, in VH) may offer new perspectives on the roles of sensory pathways and neurocognitive processes in generating hallucinations.

I begin by drawing out some candidate mechanisms identified in research into specific hallucination modalities and examining whether they might be generalizable across the range of modalities. I then consider some processes that seem likely to have relevance only for specific modalities, before considering how the notion of functional systems (Luria, 1965) might help us to reconceptualize the relations between modality-general and modality-specific processes in hallucinations. Finally, I consider some issues of clinical and diagnostic significance. My focus is on cognitive and neural processes implicated in the experience of hallucination (particularly in psychosis, although with relevance for the range of other disorders in which hallucinations occur) and in hallucinations that have no clinical significance. Factors such as genetics, social adversity, trauma, and culture – strongly implicated in the aetiology of hallucinations – are beyond the scope of this article.

## Misattribution theories

In a dominant model of AH, inner speech is misattributed to an external source, resulting in the experience of an alien voice (Feinberg, 1978; Bentall, 1990; Frith, 1992). This ‘inner speech misattribution’ model has been supported by cognitive and neuroscientific studies (Ford and Mathalon, 2005; see Alderson-Day and Fernyhough, 2015, for a review). It has also been criticized for failing to distinguish between varieties of inner speech (Fernyhough, 2004; Jones and Fernyhough, 2007), and neglecting social elements of hallucinatory experience (Alderson-Day and Fernyhough, 2016).

Some authors have considered whether the inner speech misattribution model might generalize to other modalities of hallucination (Fernyhough, 2016). A modality-general process in hallucinations might comprise a bias towards misattributing aspects of internal imagery, in whatever modality it occurs, to an external source. One challenge to such an account is the very different incidence of hallucinations in different modalities. If a modality-general process was involved, why would hallucinations not be equally common in each modality? Someone liable to misattribute their own internal auditory imagery (such as inner speech) to an external source would presumably be equally likely to misattribute olfactory, visual, or tactile imagery.

At first glance, this scenario seems unlikely given the reduced incidence of hallucinations in other modalities, particularly the tactile and olfactory (McCarthy-Jones *et al*., 2017). One plausible explanation for this pattern is that sensory modalities differ in their salience, with some modalities (such as the auditory, the typical channel for linguistic communication) being particularly emotionally significant, especially with regard to their social salience (Alderson-Day and Fernyhough, 2016). A modality-general account would need to explain differing vulnerabilities across modalities, including the possibility that experiences in a particular modality might be more salient or have greater affective significance than those in others. Thinking about modality-generality in this way thus usefully highlights specific open empirical questions.

A further issue is to define what would count as the analogue of inner speech in each modality. In VHs, one could imagine that what is misattributed is visual imagery. Consistent with this view, patients with VHs in Parkinson's disease have been shown to have elevated visual imagery strength compared to controls (Shine *et al*., 2015). While some progress is being made in understanding musical hallucinations as misattributed ‘inner music’ (Fernyhough, 2016; Moseley *et al*., 2018), it is more challenging to envisage an inner tactile sense (‘inner touch’) or inner olfaction (‘inner smell’) that could be misattributed to an external agency in the case of hallucinations. While voluntary and involuntary somatosensory and olfactory imagery (in the absence of actual sensations) are plausibly involved in typical human experience, these kinds of imagery remain only weakly understood from an empirical perspective, suggesting another way in which a modality-general approach to hallucinations might stimulate research.

## Reality-monitoring processes

A related candidate for a modality-general process in hallucinations is reality monitoring, or the capacity to distinguish between internally and externally generated experiences. Such accounts have been closely associated in the AH literature with inner speech misattribution (e.g. Bentall, 1990), but are not identical with them. It is possible to imagine, for example, that inner speech could be misattributed for reasons other than a reality-monitoring problem (such as if the contents of inner speech were ego-dystonic; Stephens and Graham, 2000), or that reality-monitoring processes might not gain traction on inner speech (if the latter, for instance, had a particularly privileged position in consciousness, such that it was unlikely to be misattributed).

There is considerable evidence for reality-monitoring biases in AH in patient samples (Brébion *et al*., 2000; Waters *et al*., 2004), alongside equivocal evidence for general population samples (Larøi *et al*., 2004; Garrison *et al*., 2017). Some studies have identified similar connections between reality monitoring and hallucination proneness in VH (Brébion *et al*., 2008; Aynsworth *et al*., 2017). In terms of brain processes, a significant body of research has pointed to the role of the anterior medial prefrontal cortex (amPFC) in reality monitoring (Mitchell and Johnson, 2009; Simons *et al*., 2017). Recent research has identified a specific candidate for a brain region playing a role in reality monitoring, the paracingulate sulcus (PCS) (Buda *et al*., 2011). Garrison *et al*. (2015) found that a 1 cm reduction in left-hemisphere PCS length increased the likelihood of a schizophrenia patient having hallucinations by 19.9%. This relationship did not vary by modality, suggesting that amPFC reality-monitoring processes associated with this structural feature of the brain might represent a modality-general process in hallucinations, at least in clinical groups.

These findings also point to general developmental morphological variation as a possible candidate for a modality-general hallucination mechanism, consistent with neurodevelopmental accounts of psychotic disorders. Support for this view comes from findings of decreased sulcification (in the right hemisphere) with the addition of one modality of hallucination beyond AH, in a comparison of patients with both AH and VH with those with AH only (Cachia *et al*., 2015). Further research is needed to determine whether a general decrease in gyrification or sulcification in either hemisphere might correlate with the number of modalities in which hallucinations are experienced (including olfactory, tactile, etc.). That said, it is too early to rule out the existence of modality-specific reality-monitoring processes that interact with specific sensory pathways in different modalities (see, e.g. Moseley *et al*., 2014). Future research that disentangles modality-general and modality-specific reality-monitoring systems in the same samples would contribute greatly to our understanding of this issue.

## Social agent representations

As noted above, one criticism of the inner speech misattribution model has been that it places insufficient emphasis on social cognitive processes in AH. Fernyhough (2004) has proposed that AH, to the extent that they are misattributions of inner speech, should be socially structured (Alderson-Day and Fernyhough, 2016). Bell (Bell, 2013; Wilkinson and Bell, 2016) has suggested that AH can be best understood as aberrant activation of representations of social agents, rather than of auditory stimuli. Support for this view comes from several sources, including the incidence of AH in individuals deaf from birth (Atkinson *et al*., 2007) and the occurrence of ‘soundless voices’ (Janet, 1891). The fact that social agents can be represented in all sensory modalities raises the possibility that aberrant activation of such agent representations constitutes a modality-general process in hallucinations.

One implication of such a view might be that aberrant activation of social agent representations should influence different sensory modalities equally strongly. That is, a hallucination involving the activation of a representation of one's mother, say, should lead the individual to experience that entity in the visual (and conceivably olfactory and tactile) modalities as much as the auditory. While certain modalities might be more salient than others (and thus more likely to feature in such hallucinations; see above), the model would predict that ‘fused’ (or simultaneous multimodal hallucinations of the same entity) would be relatively common. If activation of social agent representations is a modality-general process, it is likely to trigger activations in multiple sensory modalities simultaneously, including those that are most socially salient.

The evidence does not support this view. Recently, Lim *et al*. (2016) reported a lifetime prevalence of multimodal hallucinations of 53%, higher than the 27% lifetime prevalence for unimodal hallucinations, in patients with schizophrenia spectrum disorders. However, their data do not speak to the issue of simultaneous hallucinations of the same entity in different modalities, and are thus not relevant to the question of modality-general activation of conceptual representations, including those of social agents. Greater clarity on the definition of different types of multimodal hallucinations will be a benefit to future research on this issue (Waters *et al*., 2014).

Evidence for genuine simultaneous multimodal hallucinations remains limited. An article by Hoffman and Varanko (2006) reported on three patients with fused hallucinations. A recent study of 22 patients with psychotic disorders reporting VH showed that 19 reported simultaneous multimodal hallucinations, most commonly that of an image that talked to and touched the patient (Dudley *et al*., 2018). This finding fits with a modality-general view of hallucinatory experience as resulting from the aberrant activation of particular conceptual representations (for example, activation of a representation of ‘dog’ leading to a percept of a dog in more than one modality). It is possible that fused hallucinations are for some reason under-reported, and future studies with larger samples should address this issue as a priority. Reasons why activation of social agent representations might be partial or biased in certain circumstances, thus not leading to full fused hallucinations, should also be investigated in future research. For the present, one implication that can be drawn from the apparent relative scarcity of fused hallucinations is that hallucinations are unlikely to result solely from the modality-general activation of conceptual representations.

## Processing of prediction error

In recent years, the predictive processing framework has reinvigorated hallucination research by offering an understanding of how hallucinations can be understood as perceptual hypotheses arising within a brain that predicts the consequences of its experiences, rather than passively processing information from the environment. A particularly valuable strand of research has explored how an overreliance on strong priors, combined with compromised processing of prediction error, can lead to non-veridical acceptance of perceptual hypotheses (Fletcher and Frith, 2009). Hallucinations in all modalities undoubtedly involve a complex interplay of top-down and bottom-up processes, and an increasing focus on modalities other than the auditory may prove significant for understanding these experiences across the spectrum of psychiatric disorder and beyond.

To date, research within this framework has focused exclusively on the auditory and visual modalities. With regard to AH, research with both clinical (Horga *et al*., 2014) and non-clinical (Daalman *et al*., 2012; Alderson-Day *et al*., 2017; Powers *et al*., 2017) samples points to biases towards an expectation of linguistic, meaningful percepts that trigger internally-generated representations, such as speech imagery, resulting in non-veridical speech perception. Supporting the involvement of aberrant processing of prediction error, functional magnetic resonance imaging during hallucinations demonstrates weaker sensory prediction error signals in right auditory cortex, in comparison with controls (Horga *et al*., 2014). In the visual modality, a study with early psychosis and psychosis-prone healthy individuals showed a bias towards using prior knowledge to discriminate between ambiguous visual images (Teufel *et al*., 2015).

Despite considerable enthusiasm for predictive processing as a framework for understanding hallucinations (Powers *et al*., 2016; Griffin and Fletcher, 2017), there has been little attempt to establish whether the main constituent processes – over-dominant priors and aberrant processing of prediction error – represent modality-general or modality-specific processes. With regard to priors, on the one hand these might be expected to be modality-specific: a patient might, for example, have a bias towards detecting linguistic, meaningful content in an auditory stream, but that would not necessarily generalize to detecting perceptual content in other modalities. Much hangs on the difficult question of how priors are generated in the brain's predictive system. The current understanding of the brain as a device that generates perceptual priors on the basis of perception and action, rather than at some modality-general (perhaps conceptual) levels, suggests that over-dominant priors will be a modality-specific feature of hallucinations.

It is unclear whether the same applies to the processing of prediction error. It is possible to envisage scenarios in which error processing is tied to the sensory architecture in which it occurs, or alternatively is a modality-general feature of a specific brain's structural organization or cytoarchitecture – or (perhaps most likely) is a combination of the two. Data from delusional syndromes where anomalous sensory experiences occur (e.g. delusional parasitosis or Cotard delusion) may be relevant for understanding more about how perception and belief interact in hallucinations. Teasing apart modality-general and modality-specific components of predictive processing models of hallucination is likely to stimulate informative new research directions.

## Other candidate processes

A further candidate for a modality-general process in hallucinations is intentional cognitive inhibition (the ability to willingly suppress cognitions), problems with which are purported to lead, for example, to the intrusion of remembered material into consciousness as hallucinations (Waters *et al*., 2003; Jardri *et al*., 2016). While this association has been documented for AH (Waters *et al*., 2006), there is currently no evidence for its relevance for hallucinations in other modalities, and only two studies have linked it to hallucinations in non-clinical populations (Paulik *et al*., 2007; Alderson-Day *et al*., 2019). Further research is therefore needed to establish whether intentional cognitive inhibition represents a modality-general process in hallucinations.

Another process that may constitute a modality-general process in hallucinations concerns attribution of personal agency. For example, neuroimaging findings on the role of the supplementary motor area in hallucinations point to it having a role in judgments about whether an experience was self-authored or otherwise (Raij and Riekki, 2012; Alderson-Day *et al*., 2017). This raises the possibility that judgments about authorship of experiences might be underpinned by neural mechanisms that operate across the range of sensory modalities.

The putative modality-general processes considered above are not presented as mutually exclusive mechanisms, but rather as processes that can be combined in different specific models of hallucination. In contrast to these modality-general processes, factors in hallucination proneness linked to particular peripheral sensory systems are likely to represent modality-*specific* processes. An obvious example is the kind of VH that results from vision loss and eye disease (ffytche, 2009). Research is increasingly recognizing the importance of the auditory equivalent, hearing loss, and its association with AH (Linszen *et al*., 2018). Although research on olfactory hallucinations in psychosis is very limited (Langdon *et al*., 2011), it is plausible that deficits in olfactory processing might similarly relate to hallucinations in this modality. Further research in these areas is likely to advance understanding of the role of sensory input in hallucinations in distinct modalities, including how it interacts with the brain's mechanisms for producing and revising sensory predictions.

## Network-specific processes: a functional systems approach

Hallucinations in the auditory and visual modalities are thought to relate to specific networks whose aberrant connectivity results in non-veridical perceptual experiences. In AH, considerable evidence has amassed for fronto-temporal dysconnectivity, particularly focused around left superior temporal gyrus (STG), resulting in internally generated language (inner speech) being misattributed to an external source (Alderson-Day *et al*., 2015).

In the visual modality, distinct patterns of resting-state connectivity have been observed in patients with both auditory and visual hallucinations, compared to those with AH only, particularly in networks involving the hippocampal complex (Amad *et al*., 2014). Another study found no such VH-related connectivity difference for hippocampal regions, but did demonstrate hyperconnectivity between visual cortex and the amygdala for those with both AH and VH (Ford *et al*., 2015). VHs have been associated with reduced connectivity between visual areas and the default mode network (DMN) during hallucinatory experience (Jardri *et al*., 2013).

These findings fit with what is known about the role of resting-state networks in hallucinations more generally, particularly evidence for atypical interaction between the DMN and networks linked to cognitive control and salience. Reviewing the literature recently, Alderson-Day *et al*. (2015) suggested that these findings could be understood in terms of modality-general resting networks (such as the DMN) being implicated in hallucinations along with modality-specific networks such as those underpinning internal language generation.

It has been proposed (Fernyhough, 2010; Alderson-Day and Fernyhough, 2015) that such networks can be fruitfully considered as examples of what Luria (1965) termed *functional systems*, defined as systems of hierarchically organized processes working together in shifting constellations of components, with interchangeable constituent components allowing for change in the profile of the subsystems employed in achieving a fixed task from one occasion to another.

While Luria's insights (and those of his mentor, Vygotsky) were acknowledged in early formulations of cognitive neuropsychiatry (e.g. Miller, 1986), their implications for understanding psychopathology have rarely been examined. Luria's rejection of the ‘narrow localizationism’ that characterized nineteenth- and early twentieth-century clinical neurology goes some way beyond the acknowledgement that there needs to be a cognitive level of analysis of psychopathological experiences running alongside a neural one (see, for example, summaries of the principles of cognitive neuropsychiatry in David, 1993, and Halligan and David, 2001). Some aspects of Luria's account – such as the shaping of functional systems by social developmental processes, and the need for attention to the significance of neural disruption at different stages of development – are not directly germane to the present discussion (although are highly relevant for neurodevelopmental accounts of psychopathology; see, e.g. Alderson-Day and Fernyhough, 2015). More significant for the present discussion is Luria's notion of *interfunctional relations*, whereby functional systems are created through systems at lower levels of the hierarchy ‘plugging in’ to each other in flexible ways. To give one example, self-regulatory inner speech is, in Vygotskian–Luria theory, understood as a functional system produced when language comes to be used to regulate prelinguistic cognition (Vygotsky, 1987; Fernyhough, 2010). Inner speech can itself go on to form part of a functional system at a higher level of the hierarchy, such as when it interacts with the social cognition system to create dialogic inner speech (Alderson-Day *et al*., 2016).

The neglect of this aspect of Luria's writings by cognitive neuropsychiatry and related disciplines is not my focus here (see Mecacci, 2005). Rather, I propose that Luria's insights provide a useful way of thinking about the complex relations among modality-general and modality-specific processes in hallucinations. They enable us to understand how such processes work together in accounting for different patterns of experience across hallucination modalities, and offer a helpful conceptual framework for understanding how aberrant patterns of neural connectivity can affect cognitive function.

For example, one can propose a functional system implementing a monitoring mechanism for judging the provenance (internal or external) of linguistic material, thought to be atypical in at least some forms of AHs. Such a system would encompass the inner-speech generation system of left inferior frontal gyrus (Broca's area) and STG (including Wernicke's area), along with the modality-general reality-monitoring system centered in the amPFC. Disruption to the functional system, occurring at any point along its anatomical range, would affect monitoring of internal speech, fitting with findings that signal-detection performance for auditory material can be affected by neurostimulation to the STG (Moseley *et al*., 2014).

A functional systems approach can also help us to understand how atypicalities in one part of a system can be compensated for by flexibility in its other components (Luria, 1965; Fernyhough, 2010). For example, it is possible that AH in patient groups can be distinguished from AH in individuals who do not seek clinical help (so-called ‘non-clinical’ hallucinators) in terms of two interacting processes: a modality-general reality-monitoring system in the prefrontal cortex and a modality-specific increase in resting activation in auditory areas (Garrison *et al*., 2019). Elevated baseline activation in auditory cortex areas has previously been implicated in AH (Hunter *et al*., 2006), and in the terms of the present article would count as a modality-specific process. For example, the ‘saturation hypothesis’ (Woodruff, 2004) holds that overactivation of auditory cortex draws neurophysiological resources away from the processing of external speech, accounting for findings that AHs are associated with a reduction in responsivity to external speech in relevant areas such as STG (Ćurčić-Blake *et al*., 2017). In Garrison *et al*.'s model, clinical AHs require a ‘double hit’ of hyperactivation of sensory areas combined with a prefrontal reality-monitoring deficit. In non-clinical hallucinators, hyperactivation in the relevant sensory areas is compensated for by an intact reality-monitoring system. A functional systems approach can thus help us to understand interactions among neural systems across the clinical/non-clinical divide (Waters and Fernyhough, 2019).

Another example of a functional system incorporating both modality-general and modality-specific networks is proposed to involve inner speech and social cognition networks (Fernyhough, 2010; Alderson-Day and Fernyhough, 2015). Neuroimaging evidence supports the interaction of a left-hemisphere internal language network with a right-hemisphere social cognition network, suggesting a neural substrate for the experience of conversational inner speech or inner dialogue (Alderson-Day *et al*., 2016). Disruption to an inner speech–social cognition network might result in AH with varying degrees of agentive properties, such as non-personified voices, voices with aberrant or non-veridical social representations, or ‘soundless voices’ in which a social agent is represented but there is no concomitant auditory experience.

In sum, the present approach to understanding modality-generality of hallucinations offers a way of building on existing insights about mechanism, and showing how constituent processes interact flexibly in creating overarching functional systems at a higher level of the hierarchy – as ‘networks of networks’, as well as interfunctional collaborations between component systems. The functional systems approach is distinctive from other approaches to understanding brain-wide interactions among neural systems, such as large-scale brain networks (Bressler and Menon, 2010) and graph theoretical approaches (Bullmore and Sporns, 2009). Rather than beginning with data-driven network-general analysis of nodes and hubs, it begins by postulating interactions among cognitive systems and hypothesizing, on the basis of cognitive-neuroscientific evidence, about the neural networks that might underpin those interactions. In addition, it emphasizes that these dynamic processes can only be understood within a developmental context, and highlights how they are intimately intertwined with social and cultural factors (Luria, 1965; Fernyhough, 2010).

As documented above, the functional systems approach leads to many predictions that can be tested in empirical research. Specific research priorities highlighted by this approach include testing misattribution models of hallucinations in modalities other than the auditory, determining why some modalities of experience are more salient than others in the context of modality-general processes, distinguishing modality-general from modality-specific reality-monitoring processes, clarifying the relation between activation of modality-general social agent representations and activations in distinct sensory networks, and determining the extent to which atypicalities in processing of prediction error are specific to modalities.

## Clinical and diagnostic implications

Accounting for the presence of hallucinations in all sensory modalities, and the phenomenological richness of these experiences (Woods *et al*., 2015), seems to require that models of hallucination in psychotic illness will incorporate both modality-general and modality-specific mechanisms. Given evidence that hallucinations have little diagnostic specificity, understanding phenomenological variation in terms of differing combinations of modality-general and modality-specific features may be beneficial. For example, hallucinations in psychotic disorders might combine atypicalities relating to sensory processing (audition, vision) with a modality-general reality monitoring deficit (often implicated in diagnoses of schizophrenia), while non-psychotic hallucinations may lack the latter feature.

An improved understanding of how such mechanisms interact in specific modalities of hallucination across different diagnostic categories will likely bring dividends for therapy and management. For example, an individual presenting with serial multimodal hallucinations (VH at one time, AH at another) might benefit from treatments targeted at modality-general processes, such as reality monitoring, social agent representations, etc. An individual who only experienced hallucinations in one modality might gain more benefit from therapies aimed at modality-specific processes, such as cognitive behavioral therapy, psychoeducation, or neurostimulation related to the left-hemisphere internal language system.

The higher prevalence in psychosis of hallucinations in the auditory modality (and, to a lesser extent, the visual) should not be taken as a motivation against the search for modality-general processes. Language-related hallucinations may be particularly emotionally significant for a variety of reasons, including their strong social salience (Alderson-Day and Fernyhough, 2016). Multimodal hallucinations, particularly simultaneous multimodal hallucinations of the same entity, present an interesting special case. Although apparently not as prevalent as modality-general models might predict, it is likely that they are under-reported as a function of the greater emphasis on AH (Goodwin *et al*., 1971). This interpretation is supported by evidence that even ‘classic’ AHs (hearing voices) are associated with a range of other, particularly somatic, experiences simultaneously, which may not be picked up routinely in standard psychiatric assessments (Woods *et al*., 2015). Research has emphasized the importance of individual differences in the likelihood of seeking help for hallucinations (Peters *et al*., 2017), underlining the need for detailed clinical inquiry, particularly relating to aspects of hallucination that have tended to be overlooked by empirical research but which nevertheless figure significantly in clinical neurology and other disciplines. Reporting of fused and other less typical hallucinations is likely to be influenced by social and cultural factors, particularly when spiritual and religious interpretations play a role (Fernyhough, 2016).

## Conclusions

Understanding hallucinations in terms of separable modality-general and modality-specific processes may bear fruit for our scientific understanding of these experiences and their clinical management for those who seek help. A functional systems approach offers potential for understanding how such processes interact in generating hallucinatory experience, but also how they change over time – how, for example, the experience of hearing voices might change from being a threatening, alien experience to something that is more readily accepted as being ‘of the self’. Differing configurations of modality-general and modality-specific processes might be a key to understanding phenomenological variation across hallucination modalities, which in turn might be significant for diagnosis and therapy. Thinking about how hallucinations show commonality across different sensory domains, as well as how they are distinctive within them, may prove a valuable stimulus for future research into this significant human experience.
